# Diagnostic and Therapeutic Indications of Different Types of Mandibular Advancement Design for Patients with Obstructive Sleep Apnea Syndrome: Indications from Literature Review and Case Descriptions

**DOI:** 10.3390/diagnostics14171915

**Published:** 2024-08-30

**Authors:** Antonino Lo Giudice, Salvatore La Rosa, Giuseppe Palazzo, Carmelo Federico

**Affiliations:** 1 Department of Medical-Surgical Specialties, School of Dentistry, Section of Pediatric Dentistry, University of Catania, Via Santa Sofia 78, 95123 Catania, Italy; salvo.larosa11@live.it; 2 Department of Medical-Surgical Specialties, School of Dentistry, Section of Orthodontics, University of Catania, Via Santa Sofia 78, 95123 Catania, Italy; gpalazzo@unict.it; 3 Private Practice, 95123 Catania, Italy; federicocarmelo634@gmail.com

**Keywords:** OSAS, diagnosis, MAD, decision making process

## Abstract

Background: Mandibular advancement devices (MADs) are considered a primary alternative treatment for adults with moderate to severe obstructive sleep apnea (OSA) who are unable to tolerate or do not respond to continuous positive airway pressure (CPAP) therapy, supported by substantial scientific evidence. While a range of designs and materials for MADs are commercially available, there is a lack of clear diagnostic guidelines to assist clinicians in selecting the most appropriate device based on a multidisciplinary evaluation of OSA patients. This narrative review seeks to outline the key characteristics of MADs that clinicians should evaluate during both the diagnostic and treatment phases for patients with OSA. Methods: An extensive search of academic databases was conducted to gather relevant studies that address therapeutic and diagnostic recommendations for the design and titration of MADs. The search was carried out across EMBASE, Scopus, PubMed, and Web of Science up to May 2024. From a total of 1445 identified citations, 1103 remained after duplicate removal. Based on the inclusion criteria, the full text of 202 articles was retrieved, and 70 studies were ultimately included in this review. The extracted data were organized to generate clinical insights, aimed at guiding orthodontists in optimizing diagnostic and decision-making processes for treating OSA patients with MADs. Results: The analysis led to the identification of key clinical questions that can assist orthodontists in enhancing their approach and choosing the appropriate appliance basing on the diagnosis and clinical dento-orofacial characteristics. Conclusions: Bibloc appliances could be preferred over mono-bloc devices due to the possibility of arranging the mandibular advancement according to the patient’s clinical condition and orofacial symptoms. Provisional devices could be used as screening tools to verify the patient’s adherence to the therapy. Regardless of the MAD design, type and programmed advancement, it must be under-lined that the rule of the orthodontist/dental specialist is secondary to the other sleep-medicine specialists (ORL, pulmonologist) and must be related to (1) a preliminary assessment of MAD usage (dental anatomical conditions), (2) testing a diagnostic MAD usable during a sleep examination (PSG or DISE), (3) final treatment with a definitive MAD.

## 1. Introduction

Between 9% and 38% of adults are affected by obstructive sleep apnea (OSA), a condition characterized by repeated episodes of upper airway obstruction during sleep [1]. These episodes, occurring at a frequency of five or more per hour, result from either full (apnea) or partial (hypopnea) collapse of the airway [2]. Polysomnography (PSG) is the gold standard for diagnosing OSA, as it can differentiate between central sleep apnea and obstructive apnea or hypopnea [3]. OSA severity is typically measured using the apnea–hypopnea index (AHI) and is classified as mild (more than 5 events per hour), moderate (more than 15 events per hour), or severe (more than 30 events per hour) [4]. Recurrent airway blockages can lead to significant fluctuations in intrathoracic pressure, disrupted sleep architecture, reduced oxygen saturation, increased CO2 levels, and heightened sympathetic nervous system activity [5]. These physiological disturbances can impair cognitive function, lead to hypertension, cause excessive daytime sleepiness, and, in severe cases, contribute to heart failure and stroke [6,7]. Furthermore, OSA is associated with a higher mortality risk, highlighting its potential impact on metabolic and cardiovascular health [8,9].

Managing OSA requires a multidisciplinary approach, involving specialists such as pulmonologists, neurologists, otolaryngologists, orthodontists, surgeons, and nutritionists. While continuous positive airway pressure (CPAP) is the most effective treatment, patient compliance is often limited [10,11,12]. As a result, alternative therapies and interventions are being explored. The International and European guidelines on OSA management emphasize the importance of individualized treatment approaches [3,13,14,15].

Mandibular advancement devices (MADs) offer a viable alternative to CPAP for patients with specific anatomical obstructions of the upper airway, such as in the soft palate or oropharynx [16]. These devices work by pushing the tongue and mandible forward, which widens the upper airway and decreases collapsibility. Additionally, MADs may stiffen the pharyngeal walls, enhancing airway patency in the soft palate region [17]. Therefore, in cases of mild to moderate OSA and severe cases when CPAP is not tolerated, MADs are recommended as a first-line alternative [18,19].

Over the years, numerous studies have been published regarding the characteristics and indications of MADs. Moreover, technological advancements, including 3D imaging and computer-aided design/manufacturing (CAD/CAM), are revolutionizing the production of orthodontic appliances, including MADs. These technologies facilitate a fully digital workflow, enabling precise design and prototyping.

Despite growing evidence supporting MADs in OSA management, there remains a lack of studies addressing diagnostic criteria and therapeutic indications for selecting appropriate MADs for OSA patients. This study aimed to elucidate the clinical workflow for MAD-based OSA treatment and evaluate the effectiveness and side effects of various MAD designs in alleviating OSA symptoms. This study also presents two clinical cases illustrating the application of modern digital workflows in MAD fabrication.

## 2. Materials and Methods

The present study was designed as a narrative review concerning the diagnostic and treatment clinical indications for the use of different types of MAD. A search strategy comprising all specified keywords and free-standing terms was established up to June 2024. The databases that were chosen were EMBASE, Web of Science, PubMed, and Scopus. All listed sources of evidence were checked for additional research in their reference lists. Supplementary Material Table S1 reports the strategy search that was modified for every database. We included both prospective and retrospective studies from which it was possible to extract pertinent data regarding the diagnostic and therapeutic indications for MAD design and a titration protocol that orthodontists could use in their clinical management of OSA patients. To eventually find further works, systematic reviews addressing the aforementioned subjects were also taken into consideration. Case reports, case series, opinion and text papers, conference abstracts and book chapters were excluded.

## 3. Results

**Figure 1:**
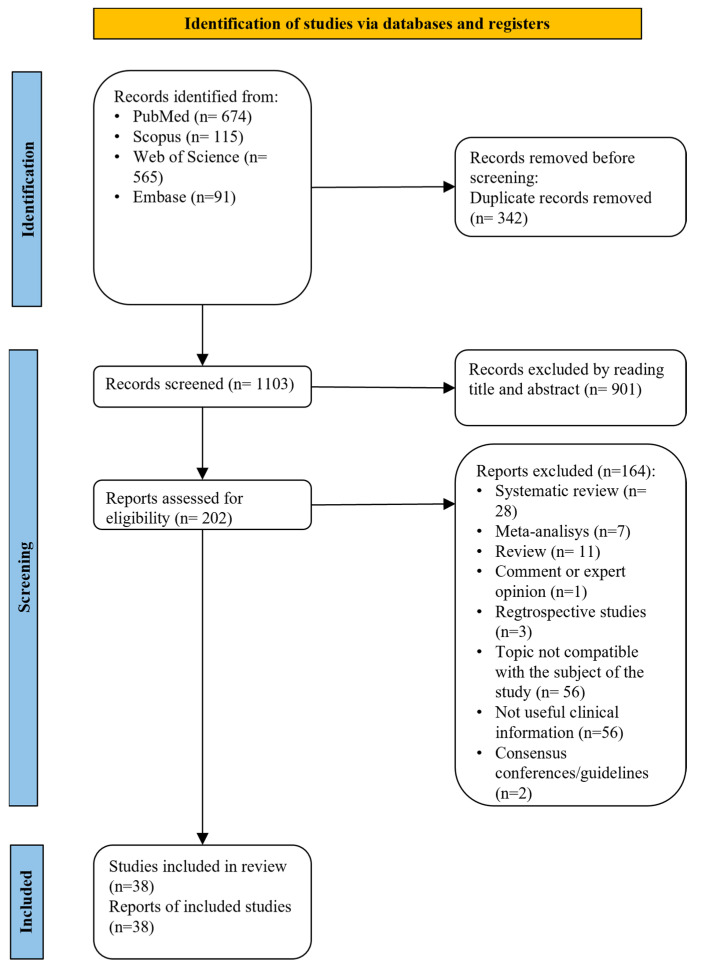
Flow chart of the included studies.

After deleting duplicate files, the reviewers examined 1103 records out of the 1445 citations found by the strategy searches. After the titles and abstracts were reviewed, 901 articles were found to be inappropriate, and the full texts of the remaining 202 papers were obtained. Following the reading of those papers as a whole, 38 studies were deemed suitable for inclusion in the current review. The papers that were excluded in this phase are listed in Supplementary Material Table S2, along with a justification for the exclusion. Figure 1 provides a summary of all of the research selection specifics and Supplementary Material Table S3 a summary of the included studies. The results were arranged and analyzed into distinct domains that comprised all of the information obtained from the included research to more effectively address the clinical indications: monobloc MAD, bibloc MAD, ready-made and custom-made MAD, MA target identification, analogic work flow, digital work flow, and hybrid work flow.

## 4. Discussion

According to the information retrieved from the available and pertinent studies in the literature, the study was organized generating specific domains elucidating all of the aspects that the orthodontist/dental specialist should consider for choosing the appropriate MAD for an individual OSA patient.

### 4.1. Query 1: Should Clinicians Use Monobloc or Bibloc MAD during Diagnostic Assessment or Treatment?

**Figure 2:**
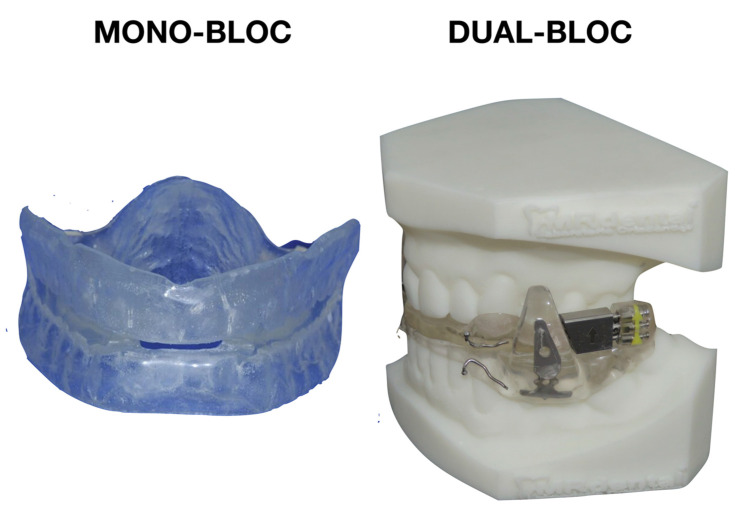
Examples of mono-bloc and bibloc mandibular advancement device.

Monobloc MADs are generally made from biocompatible materials, such as acrylic resins or thermoplastic materials, ensuring comfort and safety for the patient (Figure 2). They are single-piece devices in the mouth with a fixed MA and jaw-opening, used to enlarge the pharyngeal cross-sectional area [20]. Concerning AHI reduction, monobloc devices guarantee good effectiveness, as reported by different studies [16,21,22,23,24,25,26,27,28] in which the fixed appliance outcome was better or similar to bibloc ones. The effectiveness of monobloc design, in terms of improvements in AHI and minSaO2, was observed also in Positional-OSA (POSA) patients, and this may be explained by the fact that they are linked to a mouth-opening incapacity, which inhibits mandibular autorotation and, in turn, prevents posterior movement of the tongue base [20,25]. The fixed mandibular position could lead to a feeling of constriction when sleeping, which could result in reduced patient compliance, muscle pain (masseter), and loss of therapeutic effect [29]. Furthermore, there are numerous situations in which it is difficult to change the lower jaw’s anteroposterior posture with monoblocs [30,31]. Indeed, in a patient requiring adjustment of the MA, whether it is excessive or insufficient, it is not always easy to modify a monobloc MAD, and it may be necessary to create a new device. In addition, the need for dental technician intervention to adjust the amount of MA can cause discontinuity in treatment, recalling that constancy is crucial for patients with OSA, and it is the clinician’s responsibility to determine whether a patient might be better suited for a monobloc device.

The literature presents conflicting findings regarding patient preferences for mandibular advancement devices (MADs). Some studies suggest that monobloc devices are favored by patients, resulting in higher adherence and treatment success rates [21,30]. Conversely, other studies report the opposite [26,32,33] while two investigations concluded that there is no significant difference in patient preference between monobloc and other MAD designs [34,35]. Monobloc devices are often preferred due to their lower incidence of side effects, greater durability, simplicity of installation, and better alleviation of OSA symptoms [22]. It can be argued that for a MAD to achieve high patient satisfaction, it must not only provide therapeutic efficacy and minimize adverse effects but also ensure ease of use and longevity. Despite the perception that monobloc devices may cause significant restriction, patient preferences do not always reflect this assumption. For example, monobloc devices made of soft resin rather than hard acrylic can be installed in a single visit. Therefore, selecting the most appropriate device should take into account the individual circumstances of each patient. Just as different MAD designs can influence therapeutic outcomes, variations in patients’ anatomical and neuromuscular responses to these devices can also determine the likelihood of treatment success. In terms of side effects, monobloc devices generally produce fewer adverse outcomes compared to bibloc appliances [30,31]. For example, in Bloch’s study, monobloc devices were compared with the Herbst appliance [22]. However, studies by Serra-Torres and Yanamoto reported higher incidences of temporomandibular joint (TMJ) pain with monobloc MADs [33,36], making it difficult to definitively conclude whether monobloc or bibloc designs produce more side effects. In another study [37], side effects were evaluated in monobloc devices with varying degrees of mandibular advancement (MA). In the group with 50% MA, one patient (3%) experienced difficulty in restoring their original occlusion after therapy. After an average of three months, five patients (12%) in the 75% MA group reported TMJ pain (one resolved spontaneously, while four were switched to 50% MA). Interestingly, after six months, the 75% MA group showed a marked reduction in headaches, a benefit not observed in the 50% MA group.

**Figure 3:**
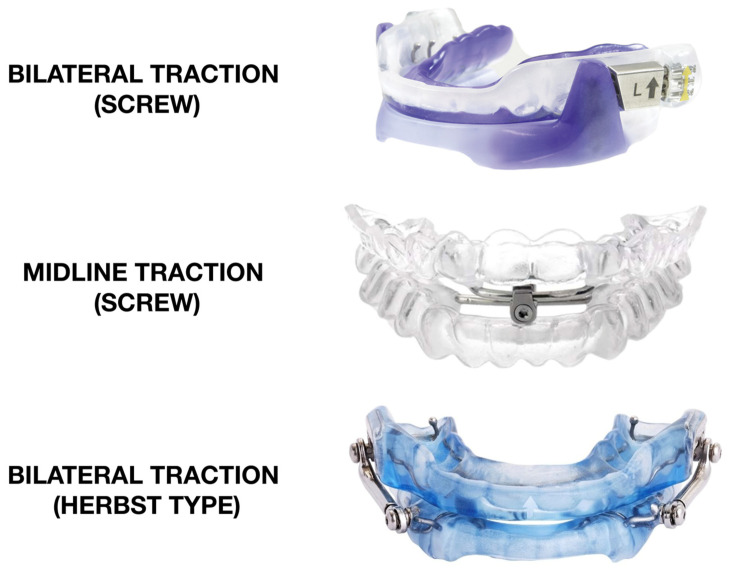
Examples of bibloc mandibular advancement devices with different types of advancement systems.

A bibloc MAD consists of two parts (blocks), one for the upper arch and one for the lower arch that may be adjusted via an intermaxillary adjustment mechanism to achieve the ideal therapeutic position (titration) by varying the degree of the mandibular protrusion (Figure 2). When worn, the device pushes the mandible into a slightly advanced position and this advancement increases the space of the upper airways, reducing the risk of obstruction that causes sleep apnea and snoring. The degree of advancement can be adjusted using a screw positioned between the upper and lower blocks of the MAD through interconnecting arms that can be modified in the laboratory, or a metal hook and base placed on the anterior maxillary and mandibular plates [38]. According to the position of the screw and the advancement mechanism, MADs can apply bilateral compression, bilateral traction (where the upper and lower part are connected in the premolar or molar zone), midline traction (where the bibloc is connected in the frontal area of the appliance) or bilateral inter-locking (without the screw) (Figure 3) [29]. Bilateral telescopic tubes can also be used to maintain the mandible’s forward posture, emulating the Herbst appliance system [38] with similar effectiveness compared to a bibloc appliance featuring a posterior titratable screw (Figure 3) [39]. 

One of the issues with bibloc devices, as previously mentioned, is the possibility of mouth opening during sleep due to the two separate blocks of the MAD, and this condition could result in a reduction of pharyngeal cross-sectional area [20]. For this reason, the usage of interarch elastics has been suggested, although studies on this topic report contrasting opinions. According to some authors [32,40,41], elastics between the upper and lower plates improve the device’s efficacy by limiting mandibular movements. However, in another study [42], no difference was found between a bibloc used with elastics and one used without.

The degree of mouth opening in bibloc devices of similar design does not have a statistically significant effect on treatment efficacy. However, devices with smaller interincisal openings tend to perform better, as an increased vertical dimension can reduce the maximum protrusive position and cause the mandible to move posteriorly or rotate backward [41,43]. In one study [41], two devices with identical designs but differing vertical openings were compared. The first device featured ball clasps embedded in the anterior aspect, secured with orthodontic elastic bands to keep the upper and lower components together during sleep. It also included removable acrylic appliances with tooth undercuts for retention, buccal flanges at an 80° angle on the lower appliance that fit against buccal blocks on the upper appliance to prevent posterior mandibular movement, and a thickness of 2.0 mm in the incisor region for both the upper and lower appliances, resulting in a 4 mm interincisal distance. In contrast, the second device incorporated an additional detachable acrylic overlay, measuring 10 mm at its thickest anterior point, placed between the upper and lower components. This overlay included retentive slots on the lower appliance with acrylic protrusions to hold it in place, increasing the interincisal distance to 14 mm. In terms of patient preference, the device with the larger interincisal opening was associated with a higher incidence of TMJ discomfort. Nonetheless, some authors have suggested that an increased vertical dimension may play a role in expanding the airway lumen by promoting greater pharyngeal wall tension and stretching [6,21,44,45].

**Figure 4:**
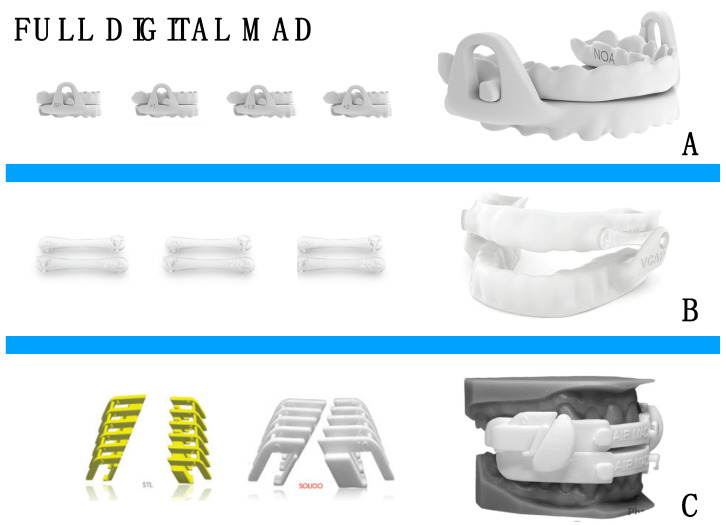
Examples of 3D printed mandibular advancement devices with different types of advancement systems. (**A**) Lower splint substitution, (**B**) lateral arms substitution, (**C**) lateral inserts substitution.

The fabrication of a custom mandibular advancement device (MAD) traditionally demands significant time and expense, necessitating multiple clinical appointments and extensive laboratory procedures. However, advancements in technology now allow for the creation of digital impressions and the development of virtual dental models, which can be accurately transferred to a virtual articulator. This process utilizes computer-aided design/computer-aided manufacturing (CAD/CAM), 3D scanning, and additive manufacturing technologies to enhance efficiency and precision. Although full-digital systems have been proposed for the fabrication of monobloc devices [46], adjustable MADs can also be produced via a digital workflow (Figure 4).

To answer Query 1, according to the available evidence, a bibloc appliance could be preferred over monobloc devices due to the possibility of arranging the mandibular advancement according to the patient’s clinical condition and orofacial symptoms. However, bibloc systems necessitate a firm control of the anterior mandibular opening via elastics or an anterior screw design. In general, the ongoing debate does not clarify which appliance (monobloc or bibloc) is superior and should represent the cold standard and further research is needed to reach a consensus on this matter.

### 4.2. Query 2: Provisional MAD and Definitive MAD. What Are the Design Characteristics, Clinical Indications and Instrumental Examinations for Their Usage?

**Figure 5:**
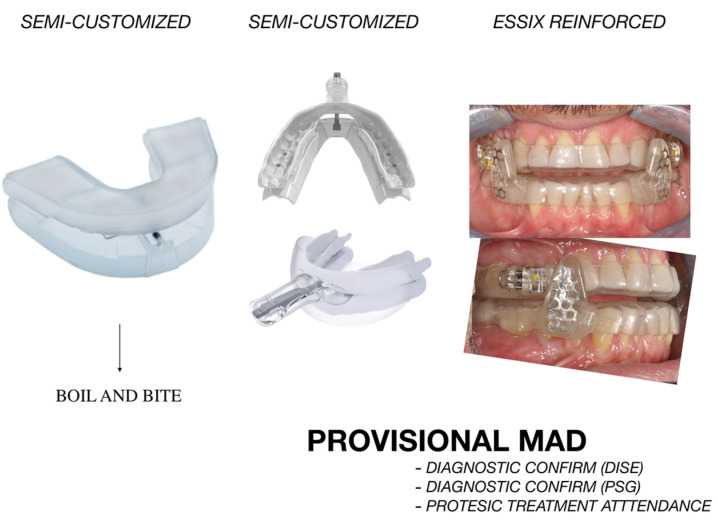
Examples of MADs for temporary diagnostic usage.

Another important aspect of MAD design is its rigidity [47]. Concerning customized fabrications, MADs are generally constructed using biocompatible resin or thermoplastic and thermoformed material. The former type provides greater rigidity and long-term integrity while thermoformed appliances can be subjected to an increased risk of breakage and are indicated for short-term or medium-term usage or diagnostic purposes. In this regard, MAD can be used as assistance in the diagnostic process in OSA subjects since sleep medicine specialists can require a PSG examination with the MAD appliance to establish the effectiveness of this treatment option. For the same purpose, a provisional MAD can be used during sleep endoscopy procedures to evaluate if the soft palate obstruction and/or retrolingual obstruction improves with the appliance in place. In such circumstances, provisional MAD can represent the appropriate choice and can be replaced by a definitive appliance after positive responsiveness is established by PSG or sleep endoscopy (Figure 5).

Non-customized appliances represent an alternative to laboratory-generated devices for provisional usability. In this regard, non-customized MADs are sold in standard sizes and shapes and were found to achieve clinical improvements in mild-to-moderate OSA [17] and are cost-effective, but Braen et al. [48] found that a custom-made MAD was more effective than a ready-made version in the management of sleep-disordered breathing. However, the main limitation of non-customized appliances is the absence of tooth-borne anchorage. In this regard, it should be remembered that if the device is not secured to the teeth enough, it may annoy the patient and result in breathing difficulties and eventually arousal. Secondly, there is no possibility of adjustment on this device. As a result, it is necessary to determine the lower jaw’s ideal protrusion throughout a maximum of two fitting procedures. For patients and doctors alike, an adjustable thermoplastic positioner might combine the benefits of a “boil-and-bite” device with a screening tool (Figure 5).

Custom-made MADs, on the other hand, have an ideal mouth fit and the option to allow the patient to gradually move their jaw forward, which increases their efficacy (Figure 2) [49,50,51]. As a result, patients are probably more comfortable and use them more frequently to manage their OSA condition [48]. However, due to the requirement that these devices should be built and fitted by a trained dentist with laboratory support, their initial cost is higher [17]. One study [52] reported that for ready-made and custom-made appliances, respectively, a full response was provided in 24% and 64% of cases. The latter research ascribed its results to the longer treatment duration and MAD design choice. Concerning side effects, the custom-made group reported much more muscle aches (14.3% vs. 5.4%) in the first two weeks. In contrast, the ready-made group reported much more discomfort from the device’s encumbrance in the mouth (19.6% vs. 4.1%), excessive salivation (14.1% vs. 2.0%), and the gag reflex (5.4% vs. 0%). During the first two months of treatment, 77.6% of patients with custom-made and 87.0% of patients with ready-made device reported discomfort at least once. After this period, there was a significant drop in the number of patients experiencing discomfort: in the first group, it dropped by 16.3%, and in the second group, by 21.8%. In general, the most often mentioned cause of discomfort and non-adherence to the ready-made devices (for this reason) was the inability to retain the device [51].

To answer Query 2, according to the available evidence, the non-customized devices could be used as screening tools to verify a patient’s adherence to the therapy [48,53]. However, to achieve greater efficacy and better patient compliance, which is crucial, it is essential to use a custom-made adjustable device that allows for advancement adjustments either in the clinic or at home by the patient.

### 4.3. Query 3: Which Extension of Mandibular Advancement Should Be Registered and Sent to the Laboratory Technician for Producing MAD?

One of the primary clinical issues for orthodontists treating patients with OSA is aiming for optimal mandibular advancement, as it is still unclear if the degree of advancement and treatment effectiveness with MADs are correlated in a dose-dependent manner [37,54,55,56]. Some studies [54,56,57,58,59] compared the effectiveness of different targeted protrusions for MAD application and found that it was more effective at 75% maximum advancement; however, the differences were not statistically significant when compared to 50% advancement, particularly in mild obstructive sleep apnea syndrome (OSAS). Moreover, other studies [60,61,62,63] evaluated the efficacy of subjective versus objectively guided titration and found that it was equivalently effective in terms of somnographic variables when using either nocturnal PSG [63], drug-induced sleep endoscopy (DISE) [60,61] or oximetry as a predictive tool [62]. Thus, at least 50% of the study group saw a significant improvement or normalization of PSG indices (AHI, ODI, and RDI) at 70% to 80% of maximum mandibular protrusion. The majority of protrusion positions were found to be 75% in the few studies comparing treatment effectiveness; however, the difference with the intermediate position (50%) was negligible and might serve as a cut-off for treating mild OSAS (50% protrusion) and moderate OSAS (75% protrusion) [14,56].

The therapeutic mandibular advanced position can be reached via subjective titration, in which the patient is gradually given more mandibular advancement over weeks until their symptoms improve or go away, or until they are unable to take any more advancement. Instead, with the objective guided titration, the therapeutic mandibular position is set a priori using the advancement that has been shown to eliminate respiratory events or non-physiologic characteristics during the diagnostic instruments/procedures (DISE, PSG, and oximetry). Objective-guided titration is more reproducible and may prevent overextension of the mandible, which subjective titration may cause. This discomfort may lead to a decrease in treatment adherence. However, because there is no option for gradual adaptation, discomfort may also arise at the beginning of treatment when the appliance is made with the target mandibular position. Clinically speaking, the titration methodology that is considered to be the gold standard may involve a methodical assessment of the therapeutic mandibular progress that may be attained by a customized titration protocol. In addition to the design, numerous studies have focused on the side effects of these devices [44,58] and the titration protocol [58] to be followed to improve symptoms without compromising patient compliance. Excessive mandibular advancement (MA), while it can further enlarge the volume of the upper airways, may also exacerbate side effects such as temporomandibular joint (TMJ) pain and muscle fatigue upon awakening.

To answer Query 3, according to the available evidence, clinicians should take the bite registration with the mandibular advancement set at least to 50% followed by a graduated titration protocol to reach the most tolerated therapeutic (advanced) position of the mandible.

Regardless of the MAD designs, types and programmed advancement, it must be underlined that the rule of the orthodontist/dental specialist is secondary to the other sleep-medicine specialists (ORL, pulmonologist) and must be related to (1) a preliminary assessment of MAD usage (dental anatomical conditions), (2) testing a diagnostic MAD usable during a sleep examination (PSG or DISE), (3) final treatment with a definitive MAD.

### 4.4. Examples of Digital Work Flow Used during Dental Diagnostic and Therapeutic Processes for Treating Adult Patients Affected by OSA with MAD

**Figure 6:**
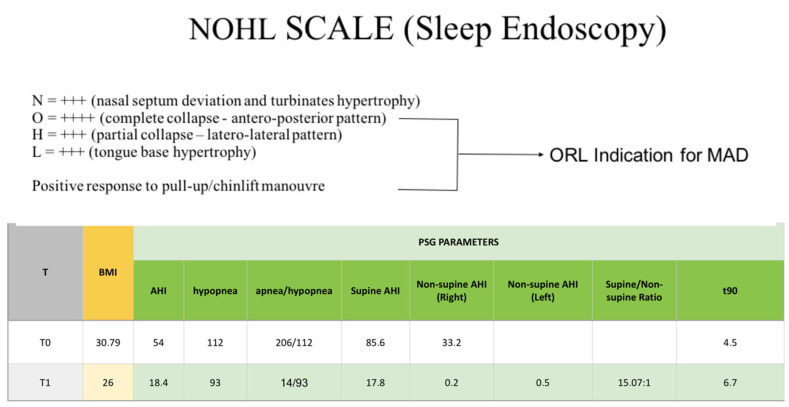
Sleep endoscopy outcomes and indications for MAD (upper), pre-treatment and post-treatment PSG parameters (lower).

- Case 1: A forty-nine y.o. man received a diagnosis of severe OSA (AHI = 54) and refused to use a CPAP (Figure 6). The patient was referred to the ORL specialist and underwent drug-induced sleep endoscopy (DISE) to better address the pattern of obstruction. The DISE revealed that the pattern of obstruction featured a complete antero-posterior collapse of the oropharynx; there was also a significant improvement of the respiratory performance (complete reduction of the apnea episodes) with the pull-up/chin lift maneuver.

**Figure 7:**
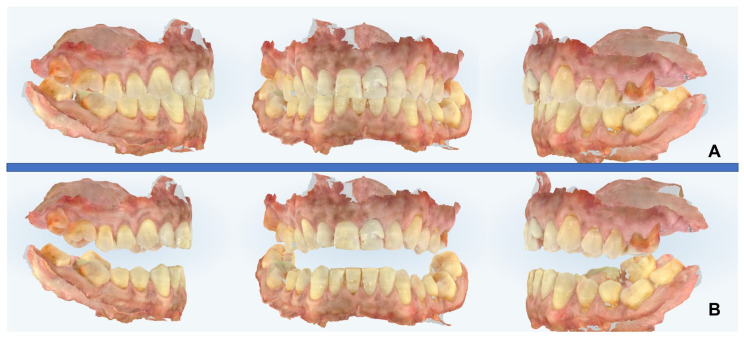
Digital intra-oral scans for MAD production. (**A**) Centric occlusion, (**B**) 50% of maximum patient’s protrusion.

**Figure 8:**
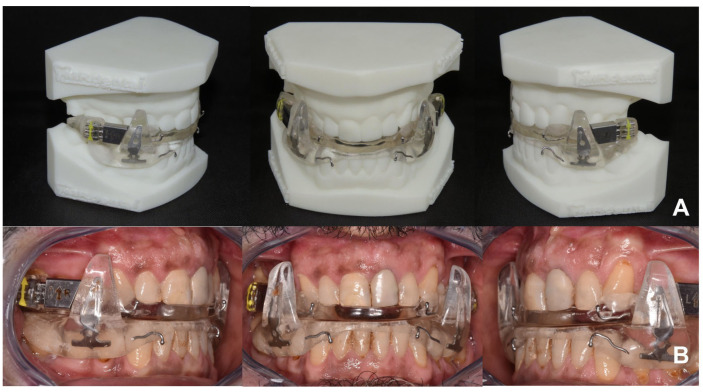
Bibloc device with posterior advancement units. (**A**) Dental cast view, (**B**) intra-oral view.

**Figure 9:**
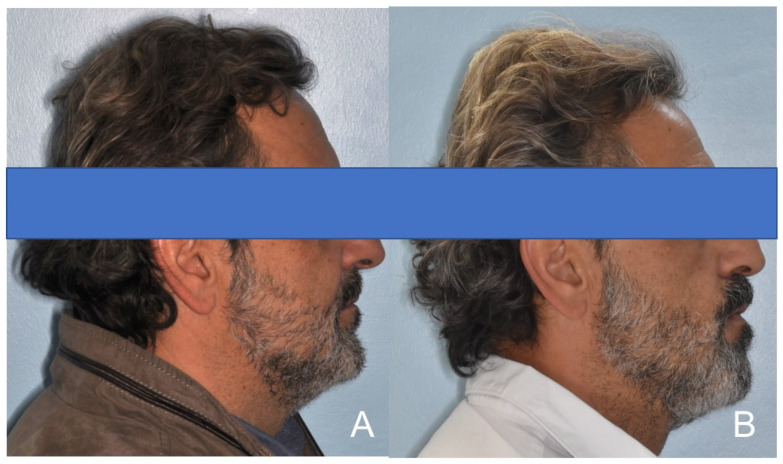
Patient profile. (**A**) Normal profile, (**B**) patient while wearing MAD.

At this time, the ORL referred the patient to a private orthodontic practice in XXX to start treatment with MAD. The advancement bite was obtained via a digital scan using a protrusive gauge (Figure 7) and the appliance was realized with a mandibular advancement of 50% of the maximum patient’s protrusion (Figure 8 and Figure 9). The titration protocol involved 0.5 mm of advancement per week until 80% of tolerated advancement was achieved. Afterward, the patient underwent a second PSG with the MAD in situ and the exam showed a significant improvement in AHI values (pre-treatment AHI = 54, post-treatment AHI = 18.4). The treatment success could be attributed to the effectiveness of the MAD and also to a reduction of the patient’s BMI (dietary regimen).

**Figure 10:**
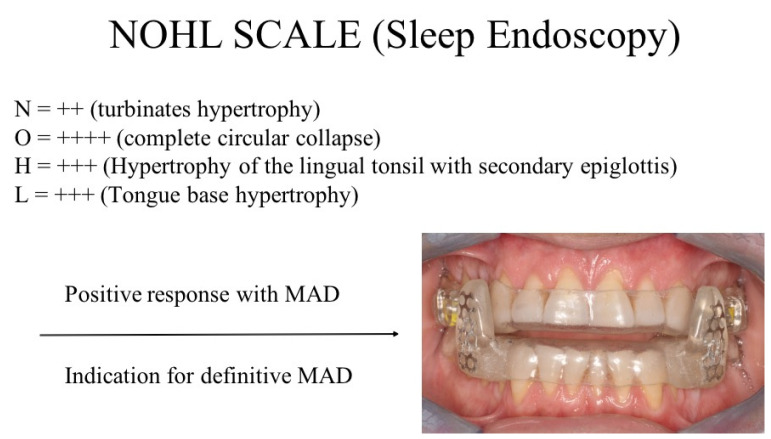
Sleep endoscopy outcomes were obtained using provisional diagnostic MAD.

- Case 2: A fifty-four y.o. man received a diagnosis of severe OSA (AHI = 35) and refused to use a CPAP. The patient was referred to the ORL specialist and underwent drug-induced sleep endoscopy (DISE) to better address the pattern of obstruction (Figure 10).

**Figure 11:**
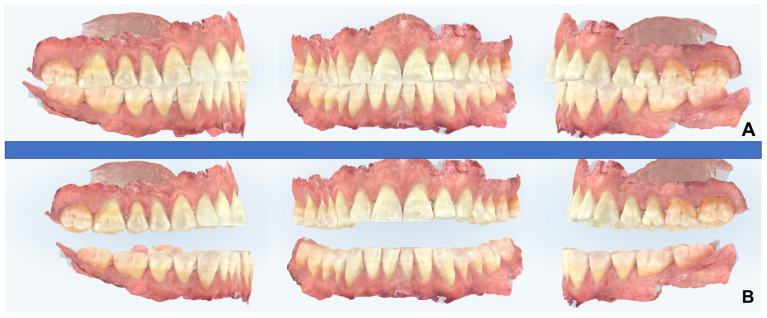
Digital intra-oral scans for MAD production. (**A**) Centric occlusion, (**B**) 70% of maximum patient’s protrusion.

**Figure 12:**
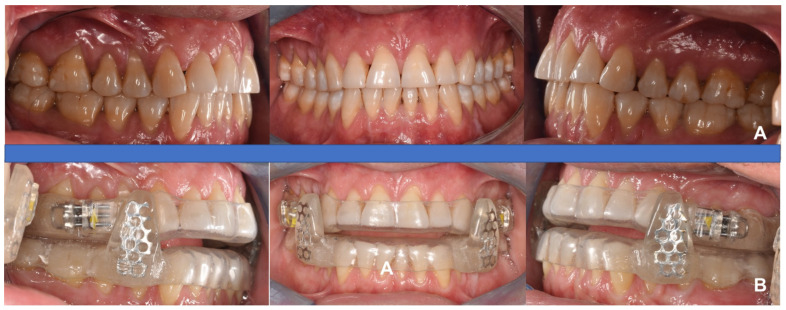
Bibloc provisional Essix-type device with posterior advancement units. (**A**) Patient’s occlusion, (**B**) intra-oral view with MAD in situ.

**Figure 13:**
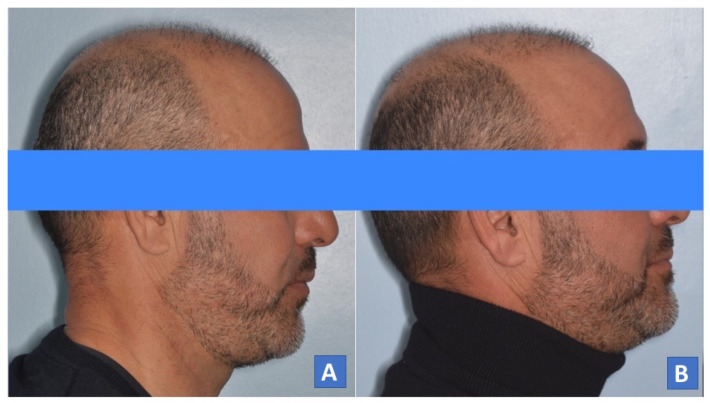
Patient profile. (**A**) Normal profile, (**B**) patient while wearing MAD.

Before performing DISE, the ORL specialist referred the patient to a dental specialist to evaluate the possibility of using MAD and to obtain a provisional diagnostic MAD usable during sleep endoscopy. The advancement bite was obtained via a digital scan using a protrusive gauge (Figure 11) and the appliance was realized with a mandibular advancement of 70% of the maximum patient’s protrusion (Figure 12 and Figure 13).

However, the ORL was instructed to advance the lower splint via screw activation in case it was necessary to increase the amount of mandibular protrusion. The provisional bibloc device was made of thermoplastic material reinforced with conventional dental resin for orthodontic appliances (Figure 12). The DISE revealed that the pattern of obstruction featured a complete circular collapse of the oropharynx associated with tongue base hypertrophy. The examination with MAD revealed a significant improvement in respiratory performance (significant reduction of the apnea episodes). At this time, the ORL referred the patient to a private orthodontic practice in XXX to continue treatment with a definitive MAD. Afterward, the patient underwent a second PSG with the MAD in situ and the exam showed a significant improvement in AHI values (pre-treatment AHI = 35, post-treatment AHI = 12.4). The treatment success could be attributed to the effectiveness of the MAD and partially to the surgical treatment of the nasal resistance (turbinates hypertrophy). However, the patient was again referred to the ORL specialist since the residual OSA could require pharyngeal surgery since the MAD was not able to completely compensate for the complete circular collapse of the oropharynx.

## 5. Conclusions

According to the available evidence in the literature, the following clinical diagnostic criteria may be drawn for choosing the appropriate MAD for an OSA patient:

- Bibloc appliances could be preferred over mono-bloc devices due to the possibility of arranging the mandibular advancement according to the patient’s clinical condition and orofacial symptoms. However, bibloc systems necessitate a firm control of the anterior mandibular opening via elastics or an anterior screw design.
- Non-customized devices could be used as screening tools to verify the patient’s adherence to the therapy. However, to achieve greater efficacy and better patient compliance, which is crucial, it is essential to use a custom-made adjustable device that allows for advancement adjustments either in the clinic or at home by the patient.
- Clinicians should take a bite registration with the mandibular advancement set to at least 50% followed by a graduated titration protocol to reach the most tolerated therapeutic (advanced) position of the mandible.

## Data Availability

Data are available upon request to the corresponding author.

## Supplementary Materials

The following supporting information can be downloaded at: https://www.mdpi.com/article/10.3390/diagnostics14171915/s1. Table S1: Terms used in database search; Table S2: Articles excluded after full-text evaluation, with reasons (*n* = 132); Table S3: Characteristics of the included primary studies.
