# Supplementary material for: Diagnostic and Therapeutic Indications of Different Types of Mandibular Advancement Design for Patients with Obstructive Sleep Apnea Syndrome: Indications from Literature Review and Case Descriptions
Source: Diagnostics (Basel). 2024 Aug 30;14(17):1915. doi: 10.3390/diagnostics14171915 (PMC11394322; doi:10.3390/diagnostics14171915)
Supplement: Supplementary file 1 [file diagnostics-14-01915-s001.zip › diagnostics-3169380-supplementary.pdf]

Supplementary Table S1. Terms used on database search.

Database	**Search format**
**PUBMED**	((("sleep apnoea"[All Fields] OR "sleep apnea syndromes"[MeSH Terms] OR ("sleep"[All Fields] AND "apnea"[All Fields] AND "syndromes"[All Fields]) OR "sleep apnea syndromes"[All Fields] OR ("sleep"[All Fields] AND "apnea"[All Fields]) OR "sleep apnea"[All Fields]) AND "syndrome*"[All Fields]) OR ("exp"[All Fields] AND ("sleep apnoea"[All Fields] OR "sleep apnea syndromes"[MeSH Terms] OR ("sleep"[All Fields] AND "apnea"[All Fields] AND "syndromes"[All Fields]) OR "sleep apnea syndromes"[All Fields] OR ("sleep"[All Fields] AND "apnea"[All Fields]) OR "sleep apnea"[All Fields]) AND ("obstruct"[All Fields] OR "obstructed"[All Fields] OR "obstructing"[All Fields] OR "obstruction"[All Fields] OR "obstructions"[All Fields] OR "obstructive"[All Fields] OR "obstructs"[All Fields])) OR (("upper"[All Fields] OR "uppers"[All Fields]) AND ("airway resistance"[MeSH Terms] OR ("airway"[All Fields] AND "resistance"[All Fields]) OR "airway resistance"[All Fields]) AND ("sleep apnoea"[All Fields] OR "sleep apnea syndromes"[MeSH Terms] OR ("sleep"[All Fields] AND "apnea"[All Fields] AND "syndromes"[All Fields]) OR "sleep apnea syndromes"[All Fields] OR ("sleep"[All Fields] AND "apnea"[All Fields]) OR "sleep apnea"[All Fields]) AND "syndrome*"[All Fields]) OR ("OSA"[All Fields] OR "OSAS"[All Fields]) OR ("snoring"[MeSH Terms] OR "snoring"[All Fields] OR "snore"[All Fields] OR "snored"[All Fields] OR "snores"[All Fields] OR (("primaries"[All Fields] OR "primary"[All Fields]) AND ("snoring"[MeSH Terms] OR "snoring"[All Fields] OR "snore"[All Fields] OR "snored"[All Fields] OR "snores"[All Fields]))) OR ("DISE"[All Fields] OR (("sleep"[MeSH Terms] OR "sleep"[All Fields] OR "sleeping"[All Fields] OR "sleeps"[All Fields] OR "sleep s"[All Fields]) AND ("endoscopie"[All Fields] OR "endoscopy"[MeSH Terms] OR "endoscopy"[All Fields] OR "endoscopies"[All Fields] OR "endoscopy s"[All Fields])) OR ("Drug-Induced"[All Fields] AND ("sleep"[MeSH Terms] OR "sleep"[All Fields] OR "sleeping"[All Fields] OR "sleeps"[All Fields] OR "sleep s"[All Fields]) AND ("endoscopie"[All Fields] OR "endoscopy"[MeSH Terms] OR "endoscopy"[All Fields] OR "endoscopies"[All Fields] OR "endoscopy s"[All Fields])))) AND ((("mandibular advancement"[MeSH Terms] OR ("mandibular"[All Fields] AND "advancement"[All Fields]) OR "mandibular advancement"[All Fields]) AND "device*"[All Fields]) OR (("mandibular advancement"[MeSH Terms] OR ("mandibular"[All Fields] AND "advancement"[All Fields]) OR "mandibular advancement"[All Fields])

	AND "appliance*"[All Fields]) OR (("mouth"[MeSH Terms] OR "mouth"[All Fields] OR "oral"[All Fields]) AND "appliance*"[All Fields]) OR "splint*"[All Fields] OR (("appliance"[All Fields] OR "appliance s"[All Fields] OR "instrumentation"[MeSH Subheading] OR "instrumentation"[All Fields] OR "appliances"[All Fields]) AND "design*"[All Fields]) OR ((("Mono-bloc"[All Fields] OR "Monobloc"[All Fields]) AND "bibloc"[All Fields]) OR "bi-bloc"[All Fields])) AND ("Randomly"[All Fields] OR ("clinical trials as topic"[MeSH Terms] OR ("clinical"[All Fields] AND "trials"[All Fields] AND "topic"[All Fields]) OR "clinical trials as topic"[All Fields] OR "trial"[All Fields] OR "trial s"[All Fields] OR "trialed"[All Fields] OR "trialing"[All Fields] OR "trials"[All Fields]) OR ("random allocation"[MeSH Terms] OR ("random"[All Fields] AND "allocation"[All Fields]) OR "random allocation"[All Fields] OR "randomization"[All Fields] OR "randomized"[All Fields] OR "random"[All Fields] OR "randomisation"[All Fields] OR "randomisations"[All Fields] OR "randomise"[All Fields] OR "randomised"[All Fields] OR "randomising"[All Fields] OR "randomizations"[All Fields] OR "randomize"[All Fields] OR "randomizes"[All Fields] OR "randomizing"[All Fields] OR "randomness"[All Fields] OR "randoms"[All Fields]) OR ("controlled clinical trial"[Publication Type] OR "controlled clinical trials as topic"[MeSH Terms] OR "controlled clinical trial"[All Fields]) OR ("randomized controlled trial"[Publication Type] OR "randomized controlled trials as topic"[MeSH Terms] OR "randomized clinical trial"[All Fields] OR "randomised clinical trial"[All Fields]))
**EMBASE**<br><br>**via Ovid**	((sleep apnoea syndrome* or OSA or OSAS or DISE or SLEEP ENDOSCOPY or Drug-Induced Sleep Endoscopy or SNORING or PRIMARY SNORING) and (Mandibular Advancement device* or Mandibular Advancement appliance* or Oral appliance* or Splint* or Appliance design* or Mono-bloc or Monobloc or bibloc or bi-bloc) and (randomly or Trial or Randomized or Controlled clinical trial or Randomized clinical trial)).af.
**WEB OF**<br><br>**SCIENCE**	((ALL=(sleep apnoea syndrome* OR OSA OR OSAS OR DISE OR SLEEP ENDOSCOPY OR Drug-Induced Sleep Endoscopy OR SNORING OR PRIMARY SNORING)) AND ALL=(Mandibular Advancement device* OR Mandibular Advancement appliance* OR Oral appliance* OR Splint* OR Appliance design* OR Mono-bloc OR Monobloc OR bibloc OR bi-bloc)) AND ALL=(randomly OR Trial OR Randomized OR Controlled clinical trial OR Randomized clinical trial)
**SCOPUS**	(sleep AND apnoea AND syndrome* OR osa OR osas OR dise OR sleep AND endoscopy OR drug-induced AND sleep AND endoscopy OR

	snoring OR primary AND snoring) AND (mandibular AND advancement AND device* OR mandibular AND advancement AND appliance* OR oral AND appliance* OR splint* OR appliance AND design* OR mono-bloc OR monobloc OR bibloc OR bi-bloc) AND (randomly OR trial OR randomized OR controlled AND clinical AND trial OR randomized AND clinical AND trial)

Supplementary Table S2. Articles excluded after full-text evaluation, with reasons (n=132)

Article excluded	Reasons for exclusion
(Aarab 2017)	6
(Al Mortadi 2022)	6
(Alanazi 2018)	1
(Almeida 2013)	7
(Alswairki 2022)	1-2
(Andrén 2013)	7
(Araie 2018)	1-2
(Azagra-Calero 2012)	3
(Bamagoos 2019)	5
(Bamagoos 2019)	7
(Baratta 2018)	6
(Barthlen 2000)	7
(Bartolucci 2016)	1-2
(Bartolucci 2019)	1-2
(Bratton 2016)	4
(Braga 2011)	6
(Bretton 2015)	2
(Camañes-Gonzalvo 2022)	1-2
(Cavaliere 2020)	7
(Chan 2011)	7
(Chang 2023)	8
(Chung 2010)	7
(Clark 1996)	7
(Collop 1997)	6
(De Vries 2018)	1-2
(De Vries 2019)	6
(De Vries 2019)	6

(Dontsos 2021)	1-2
(Ferguson 2006)	3
(Ferguson 1997)	7
(Fernández-Sanjuán 2022)	7
(Gagnadoux 2009)	7
(Gagnadoux 2017)	6
(Galic 2016)	6
(Gauthier 2011)	7
(Glos 2016)	6
(Gotsopoulos 2004)	6
(Guimarães 2021)	6
(Hans 1997)	7
(Haviv 2014)	3
(Heinzer 2015)	6
(Hoekhema 2004)	1
(Hoekhema 2006)	1
(Hoekhema 2007)	6
(Hoekhema 2007)	6
(Hoekhema 2008)	6
(Hoekhema 2008)	6
(Hoffstein 2007)	3
(Holley 2011)	5
(Iftikhar 2013)	1-2
(Iftikhar 2017)	2
(Isono 1997)	6
(Isono 2009)	6
(Johal 2005)	7
(Johal 2011)	6
(Kastoer 2016)	1
(Knudsen 2015)	2
(Kuang 2023)	6
(Kuhn 2017)	1-2
(Kurtulmus 2009)	6
(Kyung 2005)	7
(Lavie 2003)	6
(Lee 2012)	7
(Lee 2021)	3
(Levendowski 2019)	5
(Li 2013)	1-2
(Li 2020)	1-2

(Li 2022)	7
(Lindman 2001)	3
(Liu 2001)	7
(Liu 2017)	3
(Liu 2022)	7
(Ma 2017)	1
(Manetta 2022)	3
(Marchetti 2020)	3
(Marques 2019)	7
(Martins 2018)	1
(Maurer 2007)	6
(Mecenas 2022)	1
(Mohsenin 2003)	6
(Naismith 2005)	6
(Nieto 2000)	6
(Nikolopoulou 2011)	7
(Nikolopoulou 2017)	7
(Oh 2016)	6
(Okuno 2014)	1-2
(Okuno 2016)	1
(Okuno 2016)	7
(Otsuka 2006)	6
(Patel 2019)	1
(Pattipati 2022)	1-2
(Phillips 2011)	7
(Phillips 2013)	7
(Randerath 2021)	8
(Rangarajan 2022)	1-2
(Recoquillon 2019)	6
(Riachy 2017)	6
(Robertson 2003)	7
(Rossi 2021)	1
(Rotenberg 2016)	3
(Saffer 2015)	1
(Sasao 2014)	7
(Schneiderman 2021)	7
(Schütz 2013)	7
(Schwab 2003)	6
(Schwartz 2018)	1-2
(Senaratna 2017)	1
(Shelton 1993)	6
(Sharples 2016)	2
(Silva 2021)	6

(Sivaramakrishnan 2017)	1
(Sutherland 2011)	7
(Tong 2020)	7
(Trzepizur 2009)	6
(Trzepizur 2021)	2
(Trzepizur 2021)	7
(Tsolakis 2022)	1-2
(Tsuiki 2004)	7
(Uniken Venema 2022)	6
(Van Gaver 2022)	7
(Van Haesendock 2016)	7
(Vincent 2017)	7
(Vroegop 2014)	6
(Watanabe 2004)	7
(Xia 2021)	3
(Yaggi 2010)	6
(Yoshida 2001)	7
(Yoshida 2006)	6
(Young 2008)	6
(Zhang 2009)	6
(Zhang 2019)	2
(Zhao 2013)	7
(Zhu 2015)	2

ł Reasons for exclusion: 1-Systematic Review article; 2- Meta-analysis; 3-Review or scoping review; 4-Comment or expert opinion; 5-Retrospective study; 6-Topic not compatible with the subject of the study; 7-Not useful clinical information; 8-Consensus conference/guidelines

Supplementary Table S3. Characteristics of the included primary studies

**Author/ year/ study type/ country**	**Sample**	**Age**	**type of appliance**	**OSA severity a T0**	**Measurements**	**Period**	**Results**
Aarab, 2010, RCT, Netherlands [1]	20 patients	49.5 ± 8.1	MAD, is set at a constant vertical dimension with 0%, 25%, 50%, and 75% of the maximum protrusion.	21.6±11.1 AHI	PSG, ESS	39 weeks	Compared to the 25% position, the AHI values in the 50% and 75% positions were noticeably lower.
Abd-Ellah, 2024, RCT, Egypt [2]	20 patients: 10 monobloc MAD, 10 bibloc MAD	40 ±7.5	Monobloc and Bibloc MAD	10-29 AHI	PSG	6 months	When compared to monobloc MAA, the modified bibloc MAA with elastics significantly improved upper airway measurements and whole polysomnography vital parameters in patients diagnosed with obstructive sleep apnea.
Belkhode, 2023, RCT, India [3]	40 patients	30-50	MAD and custom-made oral appliance (CMOA)	21 AHI mean	ESS, PSG	3 months	The CMOA has excellent therapeutic potential and was successful in treating moderate OSA. The MAD may be able to treat people with moderately obstructive sleep apnoea with it.
Bishop, 2014, RCT, USA [4]	24 patients	47.4 ± 2.6	Klearway and TAP3	19.3 ± 4.6 AHI	ESS, SAQLI, RSR	3 months	Neither appliance proved to be more effective than the other in any AHI classification for any variable recorded
Bloch, 2000, RCT, Switzerland [5]	24 patients	50.6±1.5	Monoblock, Herbst	26.7±3.3 AHI	ESS, PSG	5 months	For the treatment of sleep apnoea, the OSA-Herbst and OSA-Monobloc work well. More patients experienced symptom relief with OSA-Monobloc than with OSA-Herbst, and because of its easy-to-use nature, most patients chose it.
Campbell, 2009, RCT, New Zeland [6]	28 patients: 12 objective advancement, 16 subjective	49.8 ± 12.6 objective, 48.1 ± 10.6 subjective	MAD	26.5 ± 12.0 AHI objective, 25.4 ± 7.4 AHI subjective	PSG, ESS, questionnaires	6 weeks	Titratable MAD was well tolerated and, in most cases, improved or eliminated OSA in a subset of patients. Although there was an increase in device use and objective confirmation of

							efficacy after three weeks, there was no significant change in symptoms or AHI with PSG-based feedback. Neither titration technique was appreciably better than the other.
Fleury, 2004, CT, France [7]	40 patients.	57 ± 9	MAD	46 ± 21 AHI	PSG, questionnaires, ESS, VAS	18 months	The efficacy of the OA titration process is increased when the oximetric score and the patient's subjective assessment are combined.
Fransson, 2022, RCT, Sweden [8]	314 patients	55 (49;65) Non POSA and 54 (47;63) POSA	Monobloc and bibloc	29 (17;39) AHI non-POSA, 23 (14;30) AHI POSA.	night at-home polygraphic study, ESS, PGIC	1 year	The idea that individuals receiving oral appliance therapy who had POSA at baseline would respond to treatment more frequently than those who did not was disproved. On the other hand, supine AHI decreased much more in the POSA group and non supine AHI decreased significantly in the POSA group.
Geoghegan, 2015, RCT, China [9]	45 patients	52	bibloc and monobloc MADs	21.1(14.2–50.1) AHI	Lateral cephalogram, PSG, ESS	26 weeks	The most effective MAD for reducing OSA severity was monobloc. Subjective OSA indicator alterations did not show any differences. Both MADs increase upper airway patency and change the posture of the surrounding musculature, as evidenced by the significant but comparable cephalometric alterations that were seen. As a result, the various MAD design elements point to an effect on a few OSA indicators.
Ghazal, 2009, RCT,	103 patients: 51 IST, 52 TAP	55.5 ± 10.6	two MADs	32 ± 6 AHI IST, 37 ± 8 AHI TAP	PSG, ESS, PSQI, questionnaires	2 years	This study shows that, even after more than 24 months, the IST and TAP devices are both useful treatment tools

Germany [10]							for treating OSA. A return of symptoms over time or insufficient improvement in anticipated subjective symptoms could be the cause of non-compliance.
Gogou, 2022, CT, Greece [11]	50 patients: 34 DISE, 16 control	48.8±12, 3	MAD	31.7 ± 17.3 AHI	DISE, PSG, questionnaires	8 weeks	For OSA patients receiving MAD therapy, DISE offers a substantial benefit. Even individuals with moderate to severe OSA conditions can benefit from its use as a useful prediction tool in clinical practice.
Isacsson 2019, RCT, Sweden [12]	302 patients: 146 bibloc, 156 monobloc	54 (12.2) bibloc, 55 (11.4) monobloc	bibloc and monobloc MADs	27 (14.2) AHI bibloc, 25 (14.1) AHI monobloc	PSG, ESS, FOSQ	2 month s	From a short-term standpoint, both appliances produced adverse events of comparable severity and had equal beneficial effects for treating OSA.
Johal, 2017, RCT, United Kingdom [13]	25 patients	44.9 (SD 11.5)	ready-made and custom-made MADs	13.3 (10.9–25) AHI	Visi-Lab Greyflash at home, ESS, FOSQ, SF-36, OAOQ	7 month s	The study shows that a customized mandibular repositioning device is significantly more clinically beneficial in treating OSA, especially when it comes to patient compliance and tolerance.
Kato, 2000, RCT, Japan [14]	37 patients	49.0 (27.1 to 66.6)	Three MADs with 2-, 4-, and 6-mm mandibular advancement s	26.0 (11.2 to 72.0) ODI	Endoscopy, oximetry	1 week	The mandibular position has a considerable impact on both nocturnal oxygenation and pharyngeal collapsibility improvement.
Kazemeini, 2022, RCT, Belgium [15]	10 patients	48.0; 41.5; 55.6	MAD with subjective, objective PSG titration and DISE titration	21.3; 17.5; 26.8 AHI	PSG, DISE	4 month s	In this pilot randomized cross-over trial, there were no differences between subjective, DISE, or PSG titrations in terms of the best mandibular

							posture and the corresponding efficacy of MAD.
Lawton, 2005, RCT, UK [16]	16 patients	44.8 (range 24.0-68.4)	Twin block, Herbst	45.5 (29.0-68.0) AHI	Questionnaires, domiciliar sleep study, ESS, SF-36, VAS	14 weeks	For the treatment of individuals with OSA, the TB MAD is a good substitute for the Herbst MAD.
Ma, 2020, CT, China [17]	42 patients	41.5 ± 9.0	MAD	23.4 ± 11.5 AHI	Rhinospirometry, rhinomanometry, magnetic resonance imaging, home sleep testing, PSG baseline	1 year	With more severe cases of OSA, there was a greater and nonlinear effect of mandibular protrusion on the decrease of AHI by mandibular advancement devices. Every patient should have a more customized mandibular protrusion.
Makihara, 2022, RCT, Japan [18]	32 patients: 17 50%, 15 75%	62.2 ± 1.90	MAD 50 and 75% of maximum mandibular protrusion	22.3 ± 13.49 AHI	ESS, PSG	4 month s	The first therapeutic mandibular position for patients with mild to severe OSA is 50% mandibular advancement. It was proposed that variations in gender also impact the efficacy of treatment.

Marklund, 2015, RCT, Sweden [19]	91 Patients: 45 MAD, 46 placebo	49.8 (10.6) MAD, 54.1 (9.4) Placebo	MAD	15.6 (9.8) AHI MAD, 15.3 (10.5) AHI placebo	ESS, KSS, OSLER, SF-36, FOSQ, PSG	4 month s	For patients with mild to severe sleep apnoea or daytime sleepiness and snoring, an adjustable, custom-made oral device improves obstructive sleep apnoea, snoring, and potentially restless legs without affecting quality of life or daytime sleepiness.
Marty, 2017, Prospective study, France [20]	35 patients	49.6 ± 14.1	MAD	34.1 ± 18.9AHI	PSG, ESS	2 month s	Respiratory and somnolence characteristics were enhanced by this specially fitted MAD, and reaction rates were comparable to those reported in the literature with other devices.
Pepin, 2019, RCT, France [21]	198 patients: 100 TALI, 98 ONIRIS	51 [SD, 12]	heat-molded and custom-made MADs	26.6 SD 10.4 AHI	ESS, VAS, SF-12, PSG	2 month s	The custom-made acrylic MAD was not inferior in the short term to the thermoplastic heat-moulded titratable MAD in patients with OSA who refused or could not tolerate CPAP.

Petri, 2008, RCT, Denmark [22]	93 patients: 33 MAD, 30 MNA, 30 placebo	50 ± 11 MAD, 50 ± 10 MNA, 49 ± 10 placebo	MAD, MNA, placebo	39.1 ± 23.8 AHI MAD, 32.6 ± 22.0 AHI MNA, 34.3 ± 26.3 AHI placebo	PSG, ESS, SF-36, QOL	4 weeks	With regard to OSA, MAA has a great deal of positive effects, even curing severe instances. The mandibular protrusion is necessary for the desired outcome. MNA doesn't work like a placebo. Within certain groups of OSA patients, MAA might be an effective substitute for CPAP.
Pitsis, 2002, RCT, Australia [23]	23 patients	50 ± 10 (29–64)	MAD-1 and MAD-2 with 4 and 14 interincisal opening	21 ± 12 (6–47) AHI	Questionnaires, PSG, ESS	2 month s	According to this study, patient acceptance is impacted by the amount of biting opening caused by MAS, although treatment efficacy is not significantly affected.
Quinnell, 2014, RCT, UK [24]	90 patients	50.9 (11.6)	thermoplasti c 'boil and bite' device, semi-bespoke device and bespoke MAD	13.8 (6.2) AHI	PSG, ESS, FOSQ, SAQLI, SF-36, (EQ-5D-3L)	5 month s	Non-adjustable MADs are economical and produce clinically significant improvements in mild to moderate OSAS. The semi-bespoke MAD is a suitable initial option among those tested.

Rose, 2002, RCT, Germany [25]	26 patients	56.8 ± 5.2	two MADs	16.0 ± 4.4 RDI	PSG, VAS, portable somnograph	20 weeks	This study demonstrates that both of the examined appliances can be utilized as an alternate form of treatment and are successful in treating patients with mild OSA. In terms of RDI and AI, the non-retentive activator outperformed the retentive Silencor® device statistically. Variations in appliance design had an impact on the course of treatment.
Sari, 2011, CT, Turkey [26]	24 patients: 12 Klearway, 12 MAD	39 ± 4.2	KW and MAD	18, 8 ± 7, 3 AHI KW, 17.9 ± 6.8 AHI MAD	PSG, ESS	1 month	According to this study, the Klearway device outperformed the MAS appliance in treating individuals with moderate OSA. The number of high apneic episodes during sleep was found to be decreased more by an appliance that offers 85% mandibular advancement to open the upper airway than by one that offers 75%.
Segù, 2021, Prospective study, Italy [27]	40 patients	55.6 ± 12.7 3	Ready-made and custom-made MADs	26.51 ± 14.7 9	PSG	12 month s	This study demonstrated that the issue of individual responses to treatment can be avoided by incorporating a simple, low-cost trial device into the therapeutic pathway for OSAS patients.

							To put it another way, this allows for an efficient patient classification process that enables a preliminary distinction to be made between treatment responders and nonresponders.
Shi, 2023, RCT, Netherlands [28]	31 patients: 16 MAD-H and 15 MAD-S	48.5(±13.9)	MAD-H (Herbst appliance); MAD-S (SomnoDent)	16.6 (± 6.7) /h AHI	ESS, PSG, CBCT	3 month s	Regarding upper airway dimensions and respiratory parameters in individuals with mild to severe OSA, there is no discernible difference between the effects of a MAD allowing unrestricted vertical opening and limited vertical opening, within the boundaries of this study.
Suga, 2014, CT, Japan [29]	20 patients: 7 rigid, 13 semi-rigid	58.1 ± 7.6 rigid, 57.9 ± 11.4 semi-rigid	rigid and semi-rigid MAD	22.0 ± 13.8 AHI rigid, 20.5 ± 8.5AHI semi-rigid	PSG, TC	3 years	Although they impact different areas of the airway, both forms of MADs improve respiratory status.
Tegelberg, 2003, RCT, Sweden [30]	74 patients: 38: 50% MAD; 36: 75%MAD	51.8 (49.0± 54.6) group 50 - 54.4 (52.4± 56.4) group 75	MAD	16.2 (2.9) AHI 50, 18.9 (4.7) group 75	PSG	1 year	Mandibular advancement with a dental appliance effectively lowers the frequency of apneas, a measure of sleep-breathing disorder. For patients with mild to severe OSA, a more pronounced mandibular advancement did not correlate with a bigger improvement in the medical

							condition. It is advised that patients with mild to severe obstructive sleep apnea receive dental appliance treatment and not begin treatment with more than 50% mandibular advancement due to a small number of adverse events in the stomatognathic system or other issues.
Tegelberg, 2020, RCT, Sweden [31]	302 patients: 146 bibloc, 156 monobloc	55 (11.4) bibloc, 55 (10.7) monobloc	bibloc and monobloc MADs	25 (12.9) AHI bibloc, 23 (13.6) AHI monobloc	PSG	1 year	While there was a statistically bigger reduction in the AHI value with the bibloc appliance, at the 1-year follow-up, there was no significant difference between the two therapy types in terms of treating OSA. However, both types of treatments positively and considerably reduced respiratory disturbances. However, the benefit of a bigger reduction in the AHI should be weighed against the higher percentage of treatment-related adverse events and dropouts among Bibloc users.
Tsuiki, 2004, CT, Canada [32]	18 patients	45.9 (9.9)	MAD	32.5 (12.3) AHI	PSG, cephalometry	7 months	Therapy success with oral appliance therapy seems to depend on

							the degree to which the upper airways' (UA) size changes in response to mandibular advancement, in addition to anterior titration of the mandibular position to widen the UA.
Tsuiki, 2005, CT, Canada [33]	52 patients, 40 test and 12 control	45.2 ± 11.5 test, 37.1 ± 7.3 control	MAD	31.7 ± 7.5 AHI	PSG, cephalometry	6 month s	Using the DigiGraph workstation to evaluate changes in the upright mandibular position, it is possible to anticipate supine oropharyngeal expansion with dental appliance therapy. Patients with obstructive sleep apnea may benefit from oral appliances due to the dose-dependent effects of the horizontal component of upright mandibular protrusion on the supine oropharyngeal size as well as velopharyngeal enlargement.
Umemoto, 2019, CT, Japan [34]	52 patients: 23 twin-block, 29 fixed MAS	52.9 ± 10.7 twin-block, 53.8 ± 8.6 fixed	bibloc and monobloc MADs	20.6 ± 11.5 AHI twin-block, 21.4 ± 15.2 AHI fixed	PSG, ESS, cephalogram radiographs	3 month s	Because fixed oral appliances can prevent mouth opening and lessen incisal overjet, these results imply that they are superior to other treatment options for OSA.
Vanderveken 2008, RCT, Belgium [35]	35 patients	49 ± 9	Custom-made MAD, thermoplasti c MAD	13 ±11 AHI	PSG, VAS, ESS	9 month s	In this study, it was found that a thermoplastic device was not as successful in treating SDB as a custom-made device. The findings imply that neither a therapeutic option nor a

							screening method for potential candidates for mandibular advancement therapy may be employed with the thermoplastic device.
Walker-Engstrom, 2003, RCT, Sweden [36]	77 patients: 40 MA 75% and 37 MA 50%	50.4 (47.7 to 53.1) MA 75%, 54.3 (52.2 to 56.4) for the 50% MA group.	MAD 50% and 75%	47.0 (5.1) AHI MA 50%, 50.4 (4.7) AHI MA 75%	PSG, ESS, questionnaires	6 month s	The findings suggest that for certain patients with severe OSA, a dental appliance may be a viable alternative to traditional treatment.
Yanamoto, 2021, RCT, Japan [37]	15 patients	50.0 (31.5-69.0)	Semi-fixed and fixed MAD	12.5 (8.9-17.0) AHI	PSG, a portable sleep test device	10 weeks	Semi-fixed MADs are better than fixed MADs in terms of patient preference and have fewer side effects, even if both types of MADs help treat patients with OSA. For OSA, semi-fixed MADs may therefore be the best treatment option.
Zhou, 2012, RCT, China [38]	16 patients	45.23 years from 26.3 to 55.4	bibloc and monobloc MADs	26.38 ± 4.13 AHI	Questionnaires, PSG, cephalometric radiography	6 month s	When patients with OSAS decide to undergo MAD treatment , the monobloc appliance should be taken into consideration since it was found to be more effective

in lowering AI and AHI than the two-piece appliance and was also favoured by the majority of patients.
